# Evaluation of Beers Criteria Implementation in the Community Pharmacy Setting to Optimize Medication Management for Older Adults—A Pilot Study

**DOI:** 10.3390/geriatrics11010015

**Published:** 2026-01-30

**Authors:** Reza Karimi, Jason Kuan, June Kume

**Affiliations:** 1 School of Pharmacy, Pacific University, HPC-Ste 451, 222 SE 8th Avenue, Hillsboro, OR 97123, USA; 2 Albertsons Companies Inc., 13485 NW Cornell Rd, Portland, OR 97229, USA; jason.kuan@safeway.com; 3 School of Physical Therapy and Athletic Training, Pacific University, 222 SE 8th Ave, Hillsboro, OR 97123, USA; june.kume@pacificu.edu

**Keywords:** polypharmacy, potentially inappropriate medications, alternative treatments, pharmacist intervention

## Abstract

**Background/Objectives:** This pilot study aimed to evaluate the feasibility of applying the Beers criteria in the community pharmacy setting and aid pharmacists in identifying and emphasizing adverse effects from potentially inappropriate medications (PIMs) for older adults. **Methods:** We applied a single-center retrospective study to collect demographic and outcome data in order to analyze dispensed PIMs for older adults. We used an evaluation tool to compare warnings between pharmacy dispensing software and the Beers criteria. Descriptive statistics were computed via standard statistical software. **Results:** Culled from a random selection of 215 patients, the medical records from 50 subjects ≥65 years old were reviewed, including 440 of their medications. Our data demonstrated that 96% of subjects were dispensed at least one PIM, with a total of 34 different PIMs distributed at varying frequencies. A comparative analysis indicated that 74% of dispensed medications had similar, but not identical, warning profiles presented in the dispensing software and Beers criteria. Anticholinergic burden of dispensed PIMs indicated that older adults were at risk of falls and delirium. By supplementing the dispensing software with Beers criteria, we were able to create clinical communication notes for providers, patients, and pharmacy students to emphasize the role pharmacists can play to minimize PIM’s adverse effects on older adults. **Conclusions:** Our data indicates the feasibility of implementing the Beers criteria in the community pharmacy setting. Integrating the dispensing software warnings with Beers criteria created a structured intervention strategy to prevent potential adverse effects and develop clinical communication notes to emphasize a more engaging role that the community pharmacy setting can play to optimize therapeutic outcomes for older adults.

## 1. Introduction

As healthcare shifts from volume-based to value-based care, and with the rapid aging of the U.S. population, community pharmacies are well positioned to apply a more engaging patient care model to serve older adults [1,2]. According to the U.S. Census Bureau, Americans aged 65 and older increased from 12.4% to 18.0% from 2004 to 2024 (to a population of approximately 61.2 million individuals), and in 2024, the population of older adults was comparable to the population of children [1,3]. Older adults in the U.S. account for nearly 50% of hospital beds, and while many are discharged home, many others are discharged to enter other patient care settings, including skilled nursing facilities [4]. At the global level, older adults who are 65 years and older comprise 9% of the global population [5].

Older adults are the largest consumer group of prescription and nonprescription medications as a result of chronic and comorbid conditions [6]. Polypharmacy, defined as concurrent use of five or more drugs, can increase risk of adverse effects, hospitalizations, and even death [7,8]. Due to a high prevalence of multiple diseases occurring in older adults and polypharmacy, this population is disproportionately at risk to experience serious adverse effects, including falls and cognitive impairment [9,10].

Because community pharmacies can be the first patient care option and the first point of contact for medication-related issues, they are often considered an essential part of healthcare [11]. Furthermore, patients visit their community pharmacies twice as often as they visit their primary care providers, particularly in non-metropolitan rural areas [12,13]. Pharmacists are correspondingly positioned to play a larger role to assist older adults with their medication management as they are trusted practitioners who are often contacted by older adults and their caregivers for advice and/or to discuss their medication- and health-related concerns. It is of paramount importance to build upon this trusted and accessible model and assist older adults in optimizing medication use in order to improve the length and quality of their lives.

With the expanding scope of the pharmacy profession, engagement of pharmacists in medication therapy management (MTM), team-based interprofessional care, and pharmacists’ patient care process (PPCP), community pharmacies can play a critical role to transition faster from product-centered care to patient-centered care [14,15,16]. In recent years, community pharmacies in the U.S. have advanced their medication management services to support public health in the community, as is evident by them providing a wide range of preventive care, particularly, immunization services. At a community pharmacy, pharmacists not only have access to patients’ current and past medications filled throughout a particular chain, but they can also ensure that the prescribed drugs are safe and effective through a dispensing software’s Drug Utilization Review (DUR) mechanism. DUR is a patient care review process that all US licensed pharmacists are required to complete when a new prescription is filled by a pharmacist. Pharmacists apply their pharmaceutical care knowledge and medication management expertise to intervene and resolve any DUR conflicts that arise from age-related adverse effects, drug–drug interactions, drug–food interactions, drug toxicities (duplication of therapy), drug allergies, medication errors, and medication adherence.

Importantly, the American Geriatrics Society (AGS) publishes a series of criteria, known as the Beers criteria, to assist clinicians worldwide in preventing older adults’ exposure to potentially inappropriate medications (PIMs) [17]. The goals of the Beers criteria are to identify PIMs, educate clinicians and patients, and serve as a tool for evaluating the quality of care, cost, and patterns of drug use in older adults [17]. The strength and quality grading of each PIM criterion are based on the level of evidence and strength of recommendation.

Additionally, the AGS has developed a list of evidenced-based alternative treatments to assist clinicians in avoiding PIMs for older adults [18]. Widely recognized as a standardized resource, the Beers criteria is updated every 3–4 years and has the potential to serve as a valuable adjunct to the dispensing software that is uniquely applied to individual commercial pharmacies.

Our study was designed to include a physical therapist who was a certified specialist in geriatric physical therapy to optimize medication management for older adults. The physical therapist offered expertise regarding the necessity of assessing patient medication use from a clinical rehabilitative perspective, given that polypharmacy, as mentioned earlier, is a well-known risk factor for increased falls. Discussions focusing on the rehabilitative setting, in collaboration with community pharmacy professionals, emphasized the importance of a patient-focused continuum of care.

The frequency and effectiveness with which community pharmacies review the PIMs listed in the Beers criteria to assist older adults with medication management are currently unknown. In this study, we tracked how well the Beers criteria have been incorporated into a dispensing platform in a well-established national chain pharmacy system in the U.S. Through a step-wise comparison and monitoring process, this pilot study assessed the impacts of integrating the dispensing platform with Beers criteria to structure an intervention designed to assist older adults in achieving optimized therapeutic outcomes. The objectives of this study are as follows: (i) to evaluate the implementation of the Beers criteria in the community pharmacy setting; (ii) to integrate a widely adopted dispensing platform with the Beers criteria to aid pharmacists in identifying and emphasizing potential adverse effects for older adults; (iii) to generate a structured and holistic communication approach to provide recommendations to providers, education to patients, and patient cases to students to emphasize the impact of polypharmacy and the role pharmacists can play to minimize PIMs’ adverse effects for older adults.

## 2. Materials and Methods

### 2.1. Study Design and Participants

An interprofessional team (two pharmacists and one physical therapist) designed and applied a single-center pilot study project to review pharmaceutical records of dispensed medication lists for older adults within the community setting. We designed an exploratory and retrospective study in which data were collected at a single point in time. The inclusion criteria for the patient population were as follows: ≥65 years old; recorded data related to their height, weight, and blood pressure (BP); and were dispensed two or more prescribed medications. The exclusion criteria were being younger than 65-year-old and did not visit the pharmacy in the preceding 6 months. We reviewed 215 patients and each medication record underwent a manual clinical review to ensure that every patient in our database had an equal chance of being selected against predefined inclusion and exclusion criteria. Following this screening process, we utilized a census of 50 eligible subjects who met all the requirements and they were enrolled as the primary cohort for this study. This investigation was approved by Pacific University’s Institutional Review Board.

We used two distinct information resources. The first resource was a computerized cloud-based dispensing pharmacy management software (Optum Enterprise Pharmacy System, EPS, version 2025.02) utilized by at least two large chain community pharmacies in the U.S. with an extensive network of over 2000 pharmacies in addition to a number of independent pharmacies. EPS is a comprehensive patient care management system that, in a single workflow mechanism, stores and displays patient information (demographic and some health records such as BP, height, and weight) and dispenses medication. In addition, EPS assists pharmacy staff in managing regular inventory and insurance processing. EPS provides a platform to perform the required DUR as described earlier. The second resource was the Beers criteria, used to identify any warnings related to a patients’ medication [17]. We reviewed patients’ medication between 23 February 2025 and 18 August 2025, during the pharmacy’s operating hours using EPS version 2025.02, the most current version at the time. Results were analyzed in September–November 2025.

### 2.2. Outcome Measures

Two outcome measures were generated. First, we compared EPS with the Beers criteria to indicate to what extent EPS has incorporated the Beers criteria into its warning system. Second, we integrated EPS warnings with the Beers criteria and used demographic data to generate a structured clinical communication tool, SBAR. SBAR stands for situation (succinctly state the problem), background (concisely present relevant information associated with the situation), assessment (provide an analysis and consider the various options), and recommendation (recommend a specific action) [19]. The EPS and Beers warnings were combined to indicate whether the harm outweighs the benefit for the indicated patient and explore a safer alternative to achieve the desired outcome.

There are a series of available drugs that have anticholinergic action (AA) due to their wide clinical effects on different diseases. It is important to avoid these drugs, particularly when they produce cumulative anticholinergic burden (ACB) scores. Since the prevalence of polypharmacy is high among older adults, there are AA-associated risks that include increased cognitive impairment, confusion, falls, and mortality. We calculated an ACB score for each drug and a cumulative ACB score for each patient. The ACB score for each drug has been rated 0 (no anticholinergic effect), 1 (weak anticholinergic effects), 2 (moderate anticholinergic effects), or 3 (strong anticholinergic effects) [20].

### 2.3. Study Setting and Population

The study was conducted at a large chain community pharmacy geographically located in northern Oregon, USA. This study accessed the pharmaceutical records of 50 older adults who were dispensed a total of 440 prescribed medications.

### 2.4. Data Collection

We collected demographic and outcome data. As part of the extraction data, a table was generated in a Word document that included six columns. Data extraction was limited to two pharmacists, as they were the only personnel with authorized access to the pharmacy’s dispensing software. Data extraction from each older adult included multiple study characteristics/domains that included gender, age, body weight, and height; BP; current and active medication list with prescribed dose and the date the medication was filled; and EPS warning for each medication. Additional information that was added to the above extracted data included the following: the Beers warning for each medication; comparison between the EPS and Beers warnings; SBAR; and patient education. Identification of the Beers warning, SBAR, and patient education were generated on the above table by two pharmacists. A geriatrics expert in the faculty reviewed SBAR and patient education notes, and then synthesized consistent, individualized patient education for the “patient education” column. Developing SBAR, patient education, and patient cases were a holistic and comprehensive communication process that integrated information from the above six columns.

A 5-point Likert-type ordinal scale tool was developed to comparatively assess qualitative differences in the depth, explicitness, and clinical usefulness of information provided by the EPS and Beers criteria regarding potential adverse effects. Ratings were based upon a holistic assessment across multiple domains, including adverse effects, contraindications or cautions for use for older adults, toxicity, and explanatory detail. Numerical values served as ordered category labels to indicate the direction and strength, with lower scores favoring EPS (1–2) and higher scores favoring Beers (4–5), and the midpoint (3) indicating comparable, though not necessarily identical, content. Results are reported as frequencies along with the median and interquartile range (IQR) to summarize the distribution of responses for this ordinal data. To ensure an objective comparison between the EPS and Beers warnings was employed, a physical therapist conducted the data comparison. In this review, the physical therapist was not involved in the selection of study subjects, the review of any case history, nor involved in the inclusion of the EPS/DUR or Beers descriptors. This approach was designed to eliminate potential bias from pharmacists, for whom EPS and DUR processes are a standard part of daily patient care at the pharmacy.

The ACB scores were used to quantify the anticholinergic-associated adverse effects of medications. A web application, ACB calculator (https://www.acbcalc.com/), which combined two reliable resources, Anticholinergic Cognitive Burden and German Anticholinergic Burden scales, was used to calculate ACB scores for all 440 dispensed medications [21,22]. These metrics are critical for identifying potentially significant implications for health outcomes such as falls in older adults [23].

### 2.5. Statistical Analysis

Descriptive statistics were provided for demographic data including age (years), BP (systolic and diastolic, mmHg), and body mass index (BMI) for aggregated data, as well as gender. Baseline comparison between gender responses were carried out. Initially, tests were performed to determine if parametric criteria for continuous variables were met. If met, parametric testing was implemented (independent *t*-test) and if not, non-parametric testing was utilized (Mann-Whitney U).Measures of central tendency, dispersion, interquartile range (IQR), and 95% confidence intervals (95% CIs) for the mean or median were calculated. Categorical data was reported as a frequency count and percentage and normally distributed numeric data was reported as mean ± SD. Descriptive statistics were generated using Microsoft Excel and SPSS (v. 30).

## 3. Results

### Baseline Characteristics

A random selection of subjects who met the inclusion criteria resulted in the identification of 50 individuals (22 males and 28 females). Table 1 indicates demographic and baseline characteristics for dispensed prescribed medications for the focused population. During the specified timeframe in which we reviewed the subject’s medications, a median of 9 different medications per individual (IQR: 6–11) were dispensed which resulted in us reviewing a total of 440 medications (Table 1).

We found considerable variability in prescribing PIMs to older adults. Analysis of our data demonstrated that 96% of subjects were dispensed at least one PIM, with a total of 34 different PIMs (drugs or drug class) distributed at varying frequencies (a range of 2–32%) in which gabapentinoids (20%), lisinopril (20%), proton pump inhibitors (PPIs, 28%), and opioids (32%) were among the most frequently prescribed PIMs to older adults.

We were interested in evaluating whether the EPS and Beers criteria resources identified a full range of adverse effects equally or differently based on a 5-point Likert scale. Our comparison showed that 74% of PIMs had similar, but not identical, warning profiles (median 3 with an IQR 3-3). The Beers criteria identified more explicit warnings for 24% (Likert scale 4 + 5) of PIMs compared with EPS. Drugs in this ranking included ciprofloxacin, colchicine, dapagliflozin, dofetilide, empagliflozin, furosemide, levetiracetam, lisinopril, and spironolactone. On the other hand, EPS identified more explicit warnings for only two PIMs (solifenacin and tamsulosin) compared with the Beers criteria (Table 2).

In addition to the PIMs list, we identified a few drugs (indicated by an asterisk) that EPS provided strong age-related warnings but were not marked as PIMs in the Beers criteria (Table 2).

We calculated an ACB score for each of the drugs presented in Table 2. From these scores, a cumulative ACB score for each patient was calculated which indicated a range of cumulative ACB scores (0–6). A total of 38 subjects (15 males and 23 females) were dispensed a total of 85 medications that had at least one ACB score. Based on our data, the cumulative ABC score for the total study population (male and female older adults combined) was 1.96 ± 1.83 (95% CI 1.44–2.48). When assessed separately by gender, cumulative ABC scores were 1.55 (SD 1.60) for male and 2.29 (SD 1.96) for female older adults.

Effective communication with both providers and patients is essential for optimizing patient care and minimizing the risk of adverse effects. Pharmacy students should be trained to not only understand the complexity of polypharmacy and the impact PIMs have on the length and quality of life but also appreciate and empathize with older adults to address their medication needs. It is critical to create learning activities that cultivate student knowledge, attitudes, and empathy toward older adults. Since both warning resources provided adverse effects, albeit to different extents, we supplemented the dispensing software with Beers criteria and developed clinical communication notes that included recommendations for providers (SBAR), education for patients, and patient cases for pharmacy students to emphasize the impact of polypharmacy and the role pharmacists can play in minimizing adverse effects for older adults. An example of the three clinical communication notes can be found in the Supplementary Material (further inquiries can be directed to the corresponding author).

Derived from the development of SBAR and patient education materials, a list of relevant adverse effects that a timely intervention could have prevented was generated. Our data analysis indicated that the generation of both communication notes would help 94% of the patient population to avoid risk for falls, followed by helping 62% to avoid dizziness, 44% to avoid muscle pain and weakness, and 34% to avoid diarrhea (Table 3). Other preventable adverse effects such as cognitive impairment, bleeding, orthostatic hypotension, and hypoglycemia were also identified among our patient population (Table 3). In addition, through the above SBAR and patient education notes, we identified that 72% of patients (16 male and 20 female older adults) were subject to at least an alternative treatment due to the potential adverse effects presented in Table 3. It is imperative not only to avoid PIMs but also identify alternative treatments that include pharmacologic or non-pharmacologic approaches to improve health and quality of life for older adults.

## 4. Discussion

Aging is accompanied by progressive physiological and lifestyle changes over time that are often complicated adversely by acute and chronic diseases and the increased incidence of polypharmacy in older adults [24,25]. In addition, increasing longevity can introduce complex healthcare challenges unique to older adults [26].

Community pharmacy can play a critical role in public health by focusing on patient safety to ensure appropriate use of medications among older adults. It is, however, unclear how often community pharmacies review the PIMs listed in the Beers criteria to assist older adults in managing their medications. In order to evaluate whether the EPS is inherently adequate to identify PIMs and their complex interactions with other drugs taken concurrently, we compared EPS warnings with Beers warnings. Our comparison demonstrated that the majority of dispensed drugs (74%) had similar, but not identical, warning profiles. These findings should be interpreted as descriptive comparisons of informational content rather than statistically generalizable estimates, given that ratings were derived from a single, non-random cohort and based upon qualitative expert judgment.

Alerts from the EPS and Beers criteria are likely developed differently using different frameworks. While the EPS has, to some extent, used data from Beers criteria, the specific algorithms used to develop medication alerts for older adults within the EPS are not available. The Beers criteria are established by an interprofessional panel of 12 geriatric care and pharmacotherapy experts, alongside three ex-officio representatives, and utilized a rigorous systematic review and evidence-based grading strategy to identify PIMs for older adults. The above difference between the EPS and Beers criteria may explain why a further analysis of the warning discrepancies between the EPS and Beers criteria led to some differences in identifying consequences of potential adverse reactions. For instance, levothyroxine and liothyronine (thyroid hormones) raised EPS warnings that they should be used with extreme caution in geriatric patients; the Beers criteria, however, did not raise any warning. On the other hand, the Beers criteria strongly recommended adjusting the dofetilide (a class III antiarrhythmic) dose if an older adult’s creatinine clearance (CrCl) was lower than 60 mL/min. Conversely, the EPS did not raise any concern for dofetilide. Furthermore, the EPS provided strong warnings for a few drugs that were not included in the Beers criteria (Table 2). It is prudent to ensure that the dispensing software and their warning resources at community pharmacies match as closely as possible to the Beers criteria. The above differences and the fact that the majority of drugs had similar, but not identical, warnings emphasize the benefit of integrating the dispensing software’s warnings with that of the Beers warnings.

Our pilot study indicated that 34 different PIMs were dispensed over a course of 6 months to 50 older adults. A further analysis showed that 27 and 24 of the above PIMs were dispensed to male and female older adults, respectively (Table 1). Among the study population, the use of opioids (32%), PPIs (28%), gabapentinoids (20%), and the ACE inhibitor (20%) had the highest prevalence among older adults. The Beers criteria strongly recommend avoiding the use of PPIs in older adults for more than 8 weeks (due to *C. difficile*, bone loss, and the adverse effects on fractures). Our data support a cross-sectional study that indicated the use of PPIs had the highest prevalence among older adults in outpatient pharmacies [27].

When we combined the EPS with Beers criteria, we reviewed AGS evidenced-based alternative treatments as well and identified important information about minimizing potential adverse effects. For instance, for opioids use, alternative non-opioid analgesics were suggested to reduce risks of falls and fractures, delirium, and sedation, including respiratory depression and death [18]. Alternatively, one could opt for deprescribing any medications altogether and instead offering non-pharmacological approaches such as educational intervention, exercise therapy, and physical therapy [18]. For PPI agents that put older adults at risk of *C. difficile* infection, pneumonia, GI malignancies, bone loss, and fractures, one can use non-pharmacological interventions such as lifestyle changes, dietary behaviors, relaxation, and weight management to reduce a risk of acid reflux-associated symptoms [18]. Including AGS alternative treatments, as added values, in the dispensing software can promote a higher level of confidence for pharmacists to intervene and minimize adverse effects for older adults. However, it is important to discuss alternative treatments with the patient, their caregiver, and provider to ensure sound clinical judgment is used. After all, the PIM alerts are warning signals, as opposed to stop signals, and discussing alternative treatments can enhance patient education and minimize the PIM-associated adverse effects.

The updated 2023 Beers criteria included a series of tables to assist clinicians in making an informed decision to avoid medications in most circumstances for older adults [17]. It is important to emphasize that the Beers criteria serve as a framework to inform and support, rather than replace, shared clinical decision-making. These tables are highly useful and serve different clinical purposes. They range from medications considered as potentially inappropriate (Beers table 2) that is organized by organ system and therapeutic category, PIM due to drug–disease or drug–syndrome interactions that may exacerbate the disease or syndrome (Beers table 3), and medications to be used with caution (Beers table 4), to potentially inappropriate drug–drug interactions that should be avoided in older adults (Beers table 5) and medications that should be avoided or their dosages should be adjusted based on renal function (Beers table 6). We identified that 50% of the dispensed PIMs (drugs or drug classes) were listed in Beers table 3, 47% were listed in Beers table 5, and 24% were listed in Beers table 6. In the following sections we describe the important roles that the above Beers tables play in optimizing medication management for older adults within the community pharmacy setting.

Kidneys undergo natural age-related changes, both functionally and structurally, demonstrated by a reduction in the glomerular filtration rate (GFR) [28]. While GFR is a highly reliable parameter for measuring kidney function, information about kidney function through a measurement of serum creatinine is a common conventional method that assists clinicians in adjusting doses for renally eliminated drugs [29]. The Beers criteria provide important information for drugs or drug classes that are affected by the renal system based on CrCl [17]. Most outpatient pharmacies associated with hospitals have access to patient charts that include results of lab tests through an electronic health record system. There is, however, limited information in most community pharmacies about a patient’s lab tests, including renal function. We identified that 24% of the dispensed PIMs needed their doses to be adjusted based on renal function. It is important to include serum creatinine levels into the dispensing software so that older adults can achieve a better therapeutic outcome in medications that are renally eliminated.

Worldwide, approximately 30% of older adults experience falls annually [30]. The impact of polypharmacy and medications’ adverse effects on falls is also well established [9]. The AGS and British Geriatrics Society have emphasized the importance of recognizing falls as a first screening step in identifying older adults who need an intervention [31]. Recognizing falls among older adults is not a routine practice in community pharmacies. Dizziness is highlighted in the 2022 World Falls Guidelines as one of the potentially modifiable risk factors [32]. Dizziness increases with age and affects over 50% of older adults as they are at a higher risk of falls and fall-related injuries [33,34]. Combining the Beers criteria with community pharmacy’s dispensing software can assist pharmacy staff in identifying drugs that cause dizziness and falls. Our data analysis demonstrated that 94% of our study population was prone to falls and 62% to dizziness. In a study conducted in the Netherlands, pharmacists who used a simple fall screening questionnaire were able to provide a fall prevention counseling service through medication adaptation and lifestyle recommendation for their older adults [35]. The Centers for Disease Control and Prevention (CDC) has developed a program, referred to as Stopping Elderly Accidents, Deaths and Injuries (STEADI), which includes the following three core elements: screen, assess, and intervene [36]. Pharmacists can use the STEADI program to screen and educate patients about falls and fractures or alternatively recommend they contact their primary physician or another interdisciplinary healthcare team member, including physical therapist, as a follow up to identify such risks [37].

Delirium is an acute medical condition and is one of the major adverse effects associated with different classes of drugs such as anticholinergics, antipsychotics, benzodiazepines/nonbenzodiazepine, benzodiazepine receptor agonist hypnotics, corticosteroids, and opioids [17]. Of particular concern are the medications that have anticholinergic properties due to blocking acetylcholine receptors in the brain and peripheral nervous system. The risks associated with concurrent use of anticholinergic adverse effects (cumulative anticholinergic burden) are both short-term, such as dry mouth, constipation, blurred vision, and urinary retention, and long-term, such as dementia, impaired physical function and falls, and these which are common reasons for hospital admission [38]. Although the aggregated data for cumulative ACB is difficult to interpret as a result of the large variances reported, the substantial presence of polypharmacy and the common combined use of several drugs that have a moderate-to-high ACB score in the current study supports earlier findings that older adults are at a higher risk for falls, cognitive decline, and delirium [8]. Regardless of short- or long-term adverse effects, close attention to the cumulative ACB score is critical to prevent the above serious adverse effects, particularly dementia and falls.

Our study population indicated that female older adults had a lower BMI and a slightly lower BP compared with male older adults (Table 1). As indicated earlier, female older adults had a higher cumulative ACB score than male older adults. It is known that females outlive males; however, it is likely that the geriatric population will have a more balanced gender distribution in the future [39]. Further research into the impacts of polypharmacy and PIMs on male and female older adults is needed to better understand the role gender plays in optimizing medication management for older adults.

As mentioned earlier, potentially inappropriate drug–drug interactions are presented in the Beers criteria as well [17]. Undesirable drug interactions between two drugs can lead to drug toxicity or a reduced effectiveness of one or both drugs. This is of particular concern for older adults as both the pharmacokinetic and pharmacodynamic metabolism of drugs are affected in this patient population. Advanced age, multiple morbidity, and polypharmacy have been identified as three main predisposing factors that are significantly affected by drug–drug interactions that ultimately result in adverse effects for older adults [40]. Our results indicated that approximately 50% of dispensed PIMs had the potential for clinically important drug–drug interactions. Pharmacists possess comprehensive medication knowledge of the mechanisms underlying drug–drug (and drug–food) interactions and their associated adverse effects. With their unique pharmacy expertise and experience, they can assist older adults and prescribers in making safer medication use and choices.

Hepatic metabolism of drugs is affected in older adults as well. It is suggested that the hepatic size decreases with age by 20–40% and the blood flow through the liver decreases by 40–60% [40]. It is reported that the cytochrome P450 (CYP450) oxidase activity in older adults may decrease by up to 30% compared to young adults [41]. Consequently, multiple drugs that are metabolized by the liver display a different metabolic rate in older adults. While the Beers criteria occasionally refers to potential for hepatotoxicity, there is no table or information that lists medications or classes of medications affected by drug metabolism. As a result, quick access to the literature and pharmacokinetic data (clearance, half-life, AUC) can assist pharmacists in intervening and identifying concerns with drug metabolism for older adults.

Suboptimal communication in healthcare settings, whether between providers or with patients, compromises patients’ safety. However, in order to minimize desensitization to warnings or similar content referred to as “alert fatigue” among patients, communications with patients need to be optimized and quality-focused to enhance patient safety and quality of care. In the high-stress environment of a healthcare setting, including community pharmacy, effective communication has an inimitable position in patient care. SBAR is a simple structured communication framework that shares pharmacotherapeutic information in a more logical and concise manner to providers who need to make rapid and accurate decisions [42]. We developed SBAR and patient education for all 50 patients and identified that a series of adverse effects could have been mitigated for the older adults (Table 3). Many of the listed adverse effects in Table 3 are, per se, risk factors related to the cause of falls. For instance, muscle weakness is a significant predictor of falls as it may impair the ability to maintain balance, stability, and mobility [43]. Another example is diarrhea that can cause dehydration and which ultimately may lead to dizziness and orthostatic hypotension, placing older adults at greater risk for fall [44,45]. Decisions about medication safety and effectiveness should take into account a variety of factors, including the use of over-the-counter (OTC) medications. It has been reported that polypharmacy in older adults is not confined to prescription drugs, and OTC products contribute to the risks that are associated with polypharmacy as well [46]. The pharmacy’s dispensing software does not capture the use of OTC products. Consequently, we were unable to assess the potential contribution of OTC products to the observed adverse drug events for older adults. Therefore, when counseling older adults, it is imperative to review the usage of OTC medication as well as to ensure that they receive proper counseling regarding the impact of both PIMs and OTC medications.

In addition to the above communication framework, we created patient cases to deliver to pharmacy students in both didactic and experiential education. It is imperative to train pharmacy students to become confident in their knowledge, communication, and ability to actively engage in optimizing medication management for older adults. Additionally, the developed SBAR, patient cases, and patient education can be disseminated among preceptors to provide a structured training paradigm for pharmacy students at experiential sites.

Our study indicates that applying the Beers criteria to assist older adults in achieving optimized patient care at a community pharmacy is feasible. With the knowledge and expertise that pharmacists have, it is of paramount importance that community pharmacists are supported to initiate multifactorial interventions to meet older adults’ medication complexity. This support will ultimately result in meeting the quadruple aim for health services, i.e., improved population health, improved patient experiences, healthcare team wellbeing, and cost savings [47].

The CDC has stated “Effective strategies for healthy aging are needed to improve the length and quality of life of older adults, and their ability to live independently” [48]. One of the challenges that community pharmacies face is rooted in a lack of adequate staff and time. It is prudent to develop strategies that involve older adults in their care to safeguard this vulnerable population from adverse effects. Based on the current study, a series of potential strategies to assist pharmacists in meeting older adults’ medication complexity at community pharmacies have been provided (Table 4).

The strengths of our pilot study stem from an interprofessional team (physical therapy and pharmacy) that collaborated to evaluate the feasibility of applying the Beers criteria at a community pharmacy. We have suggested strategies for community pharmacies to maximize their patient care services for older adults. Limitations included the small sample of the older adults studied, which may not be generalizable to the broader population. Additionally, our study indicated 34 PIMs which do not represent the full list of PIMs. Furthermore, while we ensured that every older adult in our database had an equal chance of being selected to serve as the focus of our study, we did not use any specific sampling method. Our investigation was a pilot study assessing the feasibility of conducting a larger study to explore the geriatric resources employed by community pharmacies and to evaluate the extent to which the Beers criteria and AGS alternative treatments are integrated into their patient care.

## 5. Conclusions

Community pharmacists are impeccably positioned to use their knowledge and expertise to optimize therapeutic outcomes for older adults and provide patient education to minimize the risk of polypharmacy- and PIM-associated adverse effects. Managing multiple medications is a challenging task for older adults, particularly those who experience cognitive impairment. To identify these risks, there is a need for multi-tiered strategies to be implemented at the community pharmacy. Integrating the Beers criteria and alternative treatments with the community pharmacy dispensing software can assist pharmacists in identifying medication issues so they can intervene and ensure medication safety and effectiveness for older adults. We anticipate that our findings will encourage community pharmacists to augment their patient care intervention to safeguard this vulnerable population from both short- and long-term adverse effects. Furthermore, the development of authentic SBAR, patient education, and cases reinforces both patient-centered care and learner-centered models to optimize medication management for older adults.

## Figures and Tables

**Table 1:** Demographic and baseline characteristics for dispensed prescribed medications.

Characteristics	Total	Male	Female	*p* Value
Number of subjects	50	22	28	
Age (years) *	78 ± 8	76 ± 7	79 ± 9	*p* = 0.151
Blood pressure systolic (mmHg) *	132 ± 19	134 ± 20	131 ± 18	*p* = 0.622
Blood pressure diastolic (mmHg) *	73 ± 13	75 ± 11	71 ± 14	*p* = 0.277
Body mass index (BMI) *	27.9 ± 5.2	31.1 ± 3.8	25.4 ± 4.7	*p* < 0.001 **
Total number of reviewed drugs	440	192	248	
Total number of EPS warnings	169	72	97	
Total number of Beers warnings	143	62	81	
Total number of different dispensed PIMs	34	27	24	

* Data are presented as mean ± SD; ** Male BMI is significantly greater than female BMI (U = 102, *p* < 0.001); EPS, Optum Enterprise PharmacySystem; PIMs, potentially inappropriate medications.

**Table 2:** A list of dispensed drugs, anticholinergic burden (ACB) scores, and Likert scales that indicate to what comparable extent a warning was given by the EPS or Beers criteria for older adults.

Drugs with Age-Related Warning	ACB Score	Likert Scale
Amlodipine *	0	1
Benzodiazepine (*clonazepam* or *diazepam* or *lorazepam*)	1	3
Ciprofloxacin	0	5
Clindamycin *	1	2
Clonidine	0	3
Colchicine	0	5
Dicyclomine	3	3
Dofetilide	0	5
Doxepin	3	3
Estradiol (patch)	0	3
Furosemide	0	5
Gabapentinoids (gabapentin or pregabalin)	0	3
Hydroxyzine	1	3
Insulin	0	3
Lamotrigine	0	3
Levetiracetam	0	4
Levothyroxine *	0	1
Liothyronine *	0	1
Lisinopril	0	5
Losartan	0	3
Medrol	1	3
Metformin *	1	2
Nitrofurantoin	0	3
Non-dihydropyridine calcium channel blockers (Diltiazem or Verapamil)	0	3
NSAIDs	0	3
Opioids (Hydrocodone or *oxycodone*, or oxycontin)	1	3
Oxcarbazepine	0	3
Propranolol *	0	1
Proton pump inhibitors (*omeprazole* or *pantoprazole*)	1	3
Second-generation sulfonylureas (glimepiride and glipizide)	0	3
Selective serotonin reuptake inhibitors (*citalopram*, *escitalopram*, *fluoxetine*, and *sertraline*)	1	3
Sodium-glucose transporter 2 inhibitors (dapagliflozin and empagliflozin)	0	4
Solifenacin	3	2
Spironolactone	0	5
Statins (atorvastatin and rosuvastatin) *	0	2
Serotonin and norepinephrine reuptake inhibitors (duloxetine and *venlafaxine*)	1	3
Tamsulosin	0	2
Tramadol	2	3
Trazadone *	0	1
Trospium	3	3
Valproic acid	1	3
Zolpidem	0	3

* Age-related warning presented only in EPS; EPS, Optum Enterprise PharmacySystem; Drugs with an ACB score within a drug class are indicated in italics.

**Table 3:** Preventable adverse effects and demographic frequency (%) for total population and by gender for older adults that were identified during the creation of SBAR and patient education materials.

Preventable Adverse Effects	Total Patients N = 50	Male n = 22	Female n = 28
Potential fall risk	47 (94%)	20 (90.9%)	27 (96.4%)
Dizziness	31 (62%)	13 (59.1%)	18 (64.3%)
Muscle pain/weakness	22 (44%)	11 (50.0%)	11 (39.3%)
Diarrhea	17 (34%)	8 (36.4%)	9 (32.1%)
Cognitive impairment	14 (28%)	5 (22.7%)	9 (32.1%)
Bleeding/bruising risks	14 (28%)	8 (36.4%)	6 (21.4%)
Orthostatic hypotension	12 (24%)	3 (13.6%)	9 (32.1%)
Hypoglycemia	4 (8%)	3 (13.6%)	1 (3.6%)

**Table 4:** Suggested strategies to promote patient care for older adults at community pharmacies.

Strategy	Operation
Supplementing and/or updating dispensing software’s warnings with Beers warnings	To accurately provide pharmacy interventions to produce optimized therapeutic outcomes
Encouraging pharmacy staff to be familiar with the Beers criteria and PIMs	To make use of available resources and keep staff current with new evidence for PIMs
Permitting dispensing software to have access to patients’ serum creatinine	To recommend a dose change or alternative therapy for renally eliminated PIMs
Utilizing AGS alternative treatments	To discuss both pharmacological and non-pharmacological alternative treatments with patients, caregivers, and providers
Creating a supported staff-resourced environment to generate SBAR and patient education	To identify issues with polypharmacy and augment effective communication with patients, caregivers, and their providers
Calculating ACB score (or incorporating ACB scores into the dispensing system)	To caution patients, caregivers, and providers about delirium
Utilizing the CDC’s STEADI resources	To estimate the risk of falls and caution patients and their caregivers about falls and fractures

ACB, anticholinergic burden; AGS, American Geriatrics Society; CDC, Centers for Disease Control and Prevention; PIMs, potentially inappropriate medications; SBAR, situation–background–assessment–recommendation; STEADI, Stopping Elderly Accidents, Deaths and Injuries.

## Data Availability

Examples of patient education notes are available from the authors upon request.

## Supplementary Materials

The following supporting information can be downloaded at: https://www.mdpi.com/article/10.3390/geriatrics11010015/s1. A supplementary file that includes an example of SBAR, patient education, and patient case is enclosed.

## Abbreviations

The following abbreviations are used in this manuscript:

AGS	American Geriatrics Society
ACB	Anticholinergic Burden
ACE	Angiotensin-Converting Enzyme
BP	Blood Pressure
CDC	Centers for Disease Control and Prevention
CrCl	Creatinine Clearance
DUR	Drug Utilization Review
EPS	Optum Enterprise PharmacySystem
GFR	Glomerular Filtration Rate
MTM	Medication Therapy Management
NSAID	Non-Steroidal Anti-Inflammatory Drugs
PIMs	Potentially Inappropriate Medications
PPCP	Pharmacists’ Patient Care Process
PPIs	Proton Pump Inhibitors
SBAR	Situation–Background–Assessment–Recommendation
SPSS	Statistical Package for the Social Sciences
STEADI	Stopping Elderly Accidents, Deaths and Injuries
