# Supplementary material for: Evaluation of Beers Criteria Implementation in the Community Pharmacy Setting to Optimize Medication Management for Older Adults—A Pilot Study
Source: Geriatrics (Basel). 2026 Jan 30;11(1):15. doi: 10.3390/geriatrics11010015 (PMC12921929; doi:10.3390/geriatrics11010015)
Supplement: Supplementary file 1 [file geriatrics-11-00015-s001.zip › geriatrics-4063882-supplementary.pdf]

Supplementary Material

This supplementary material includes *deidentified* patient information. The information that included in the patient case (please see page 3, name, age, race, living situation, social history, allergies, medical history, and chief complaint) is fictional and provided for illustrative purposes only. Our research study was conducted in accordance with the Declaration of Helsinki, and approved by the Institutional Review Board of Pacific University (IRB Reference Number: IRB: 0004173, 136-24, 01/15/2025) for studies involving humans. The authors attest that Safe Harbor procedures for deidentification were used and we have removed all identifiers from the list of patient data. Further data inquiries can be directed to the corresponding author, Reza Karimi, karimir@pacificu.edu

Data Collection

We collected demographic and outcome data and implemented a structured and holistic communication approach to develop recommendations to providers (SBAR), counseling to patients (patient education), and cases to students (patient cases) to emphasize the impact of polypharmacy and the role pharmacists and community pharmacies can play to minimize potentially inappropriate medications (PIM) adverse effects for older adults (for more details, please see "Materials and Methods" section of this study). Although only 25 patient cases were fully developed, SBAR and patient education notes were created for all 50 patients. The following section is an example that describes three clinical communication notes that were developed in our study for one male patient: 1. SBAR, 2. Patient Education, and 3. Patient Case, based on demographic and outcome data.

Patient Demographics & Background

- **Name:** Hu Man
- **Age:** 78 years old
- **Sex:** Male
- Blood Pressure (BP): 124/62 mmHg
- Body Mass Index (BMI): 33.1 kg/m²

Medication Profile

Medication	Dose/Route
Glipizide ER	5 mg PO QAM
Metformin	1000 mg PO BID
Humulin KwikPen	Per sliding scale SQ
Humalog KwikPen	Per sliding scale SQ
Rosuvastatin	10 mg PO QPM
Verapamil ER	240mg PO QD
Amlodipine	10 mg PO QD
Lisinopril	10 mg PO QD
Apixaban	5 mg PO BID

1. **SBAR** (situation-background-assessment-recommendation)
    - Situation: a 78-year-old male patient with 33.1 kg/m² BMI (class 1 obesity) and normal BP; based on his medication list, most likely he is experiencing type 1 diabetes, cardiovascular issues, and high cholesterol levels.
    - Background: He is on multiple antidiabetic agents which include 2 insulin agents and 2 oral antidiabetic agents (metformin and glipizide), two different Calcium Channel Blockers (verapamil and amlodipine), one angiotensin-converting enzyme inhibitor (ACEI, lisinopril), and an anti-blood clotting agent (apixaban). He seems managing well his BP but is obese.
    - Assessment: It is unclear why he is using both Dihydropyridine Calcium Channel Blocker (DH-CCB) and Non-Dihydropyridine Calcium Channel Blocker (NDH-CCB) which may worsen heart failure (HF); it is unclear why he is using two different oral antidiabetic agents (metformin and glipizide). Per Beers criteria, sulfonylureas (glipizide) should be avoided as add on-therapy due to their association with a higher risk of cardiovascular events, mortality and hypoglycemia. The dose for the statin is considered moderate intensity, it may cause stomach upset (particularly he uses metformin) and muscle pain/weakness (particularly he uses lisinopril that may cause muscle weakness as a result of hyperkalemia). Among his medications only metformin has an anticholinergic burden score of 1 (weak anticholinergic effects).
    - Recommendation: He might benefit from using a long-acting basal insulin (such as glargine) as his current Humulin (short acting) and Humalog (rapid acting) may not be effective to manage his daily blood glucose levels. Per alternative treatment guidelines, options to replace glipizide include sodium-glucose transporter 2 (SGLT2) inhibitors and metformin, particularly if the patient has HF, cardiovascular issues, or chronic kidney disease. Continue with metformin and discontinue glipizide.

2. **Patient Education**
    - Discuss with your provider to ensure that it is necessary to use both Calcium Channel Blockers (verapamil and amlodipine). Monitor your blood pressure regularly and contact your provider if you note changes in swelling in your legs or when your blood pressure does not improve.
    - Your other blood pressure agent (lisinopril) may cause cough and/or headaches. It may cause rare but serious swelling of the face and throat. If this occurs, seek medical help immediately and notify your doctor.
    - Discuss with your provider to discontinue glipizide as this drug class (sulfonylurea) is the worst to cause hypoglycemia.
    - Use Humulin Kwikpen with extreme caution and discuss with your provider other possible medication options. It may increase risk of hypoglycemia. Know the signs of hypoglycemia (hunger, tremor, dizziness).
    - It is better to take your cholesterol reducing agent (rosuvastatin) at bed time and avoid grapefruit & grapefruit juice as they may increase the side effects. This agent may cause stomach upset. Make sure that you do not miss any follow-up tests as your provider will adjust the rosuvastatin dose or change the drug based on your liver and kidney function and your lipid panel.
    - Your balance may be affected (potential fall risk). While it is important to do daily exercise, in order to lower the chance of feeling dizzy, stand up slowly and be careful going up and down stairs and use handrails on stairs to prevent fall risk.
    - Follow salt intake advice.
    - When you use your apixaban, do not use NSAIDs like ibuprofen or naproxen, unless stated otherwise by your provider. This medication may result in bleeding and bruising.

3. **Patient Case (with answer keys):** students are expected to develop the indicated Medication-Related Problems and a SOPA (subjective, objective, assessment, plan) note.

3.1. Patient Demographics and Background

- Name: Hu Man
- Age: 78 years old
- Sex: Male
- Race: Caucasian
- Living Situation: Married, lives with spouse
- Social History:
  - Non-smoker
  - Occasional alcohol use (social drinking)
  - Sedentary lifestyle with minimal structured exercise
  - Diet high in carbohydrates and sodium
- Allergies: No known drug or food allergies
- Medical History:
  - Type 2 Diabetes Mellitus
  - Hypertension
  - Hyperlipidemia
  - Obesity
  - Venous Thromboembolism (VTE) Treatment

3.2. Chief Complaint

- "I recently got out of the hospital for a fall 3 weeks ago, caused by my low blood sugar. They told me to meet with my primary care provider soon, since there might be some things I should change about the medications I'm taking. Sometimes I get a little shaky after I take my morning pills."

3.3. Current Illness

- Diabetes: Managed with Metformin, Glipizide, Humulin, and Humalog. The patient reports they have been feeling shaky in the mornings after they take their medications. He was recently discharged from the hospital due to a fall caused by hypoglycemia. He tests his blood glucose most mornings before breakfast. He confirmed he is testing blood glucose before his glipizide dose.
- Hypertension: Currently managed by Verapamil ER, Amlodipine, and Lisinopril. His blood pressure is within guideline-recommended goals. He reports having some shortness of breath after climbing stairs or working in the yard.
- Hyperlipidemia: Managed by rosuvastatin. He reports no side effects.
- Anticoagulation: Managed by apixaban. Started due to a VTE. Reports no extreme side effects; some bruising

3.4. Medication Profile

Medication	Dose/Route	Indication
Glipizide ER	5 mg PO QAM	Type 2 Diabetes
Metformin	1000 mg PO BID	Type 2 Diabetes
Humulin KwikPen	Per sliding scale SQ	Diabetes
Humalog KwikPen	Per sliding scale SQ	Diabetes
Rosuvastatin	10 mg PO QPM	Hyperlipidemia
Verapamil ER	240mg PO QD	Hypertension
Amlodipine	10 mg PO QD	Hypertension
Lisinopril	10 mg PO QD	Hypertension
Apixaban	5 mg PO BID	Atrial Fibrillation

3.5. Vital Signs

- Blood Pressure: 124/62 mmHg
- Body Mass Index (BMI): 33.1 kg/m²
- Average Fasting BG (Patient reported): 122 mg/dL

3.6. Medication-Related Problems is used to assess how well students categorize and track patient-specific medication-related problems.

- Anticholinergic Burden: 1 (Metformin)
- Indication: Verapamil ER + Amlodipine therapy. Duplicate therapy due to the same/similar mechanism.
- Indication: Humulin + Humalog therapy. Depending on usage, it may be redundant as both are fast-acting insulins.
- Safety: Glipizide + Insulin therapy. Not indicated due to hypoglycemia risk.
- Safety: Glipizide therapy. Recommend discontinuing per Beer's Criteria due to increased risk for cardiovascular events.
- Safety: Insulin sliding scale without basal insulin. Recommend discontinuing per Beers Criteria due to hypoglycemia risk.
- Safety: Verapamil therapy. Recommend discontinuing per Beer's Criteria due to fluid retention/HF exacerbation risk.

3.7. A SOAP note (subjective, objective, assessment, plan) is used to assess students' clinical reasoning and how well they apply primary literature and guidelines to assess and develop an effective plan.

**Subjective**	• The patient is coming in for a refill of their glipizide and is complaining of occasional shakiness after taking their morning medications. He also has been having some shortness of breath with more intense daily activities.
**Objective**	• No known drug allergies<br>• Patient's blood pressure is 124/62<br>• Fasting blood glucose is averaging 122 mg/dL<br>• A full med list is available and the patient is fully adherent, receiving their medications 1-2 days early most months.

**Assessment**	• The patient's main complaints are the morning hypoglycemia symptoms and shortness of breath with some physical activities.<br>• Their blood pressure is within goal and they aren't reporting any hypotension symptoms.<br>• Their average fasting blood glucose is within goal.
**Plan**	• Discontinue: Glipizide ER<br>• Start: Insulin Glargine 10 units SQ QPM<br>• Follow-Up: 2 weeks to monitor fasting blood glucose and side effects<br><br>• Discontinue: Verapamil ER<br>• Follow-Up: 2 weeks with at-home blood pressure log to assess need for lisinopril titration
